# Non-canonical and induced neoantigens as emerging sources of cancer-specific immunotherapy targets

**DOI:** 10.3389/fimmu.2026.1876000

**Published:** 2026-07-02

**Authors:** Viacheslav V. Kudriavskii, Valeriia A. Koss, Victoria O. Shender, Georgij P. Arapidi

**Affiliations:** 1 Lopukhin Federal Research and Clinical Center of Physical-Chemical Medicine of Federal Medical Biological Agency, Moscow, Russia; 2 Biochemistry Department, Faculty of Biomedicine, Pirogov Russian National Research Medical University, Moscow, Russia; 3 Center for Bio and Medical Technology, Moscow, Russia; 4 Shemyakin-Ovchinnikov Institute of Bioorganic Chemistry of the Russian Academy of Sciences, Moscow, Russia; 5 Moscow Center for Advanced Studies, Moscow, Russia

**Keywords:** neoantigens, neoepitopes, immunotherapy, chemotherapy, splicing

## Abstract

Immunotherapy has transformed cancer treatment, yet its clinical benefit remains limited in many tumors, particularly those with low mutational burden. Because most current immunotherapeutic strategies rely on neoantigen recognition, expanding the repertoire of targetable tumor-specific antigens is essential. In this review, we discuss non-canonical and therapy-induced neoantigens – derived from alternative splicing, RNA editing, transposable elements, and aberrant translation – as emerging sources of immunotherapy targets, with emphasis on their potential to improve the efficacy of current treatment approaches. We summarize recent evidence supporting the immunogenicity of corresponding neoepitopes and highlight therapy-induced antigen generation as a promising but underexplored opportunity. In particular, we focus on the impact of splicing dysregulation and chemotherapy-induced splicing alterations on neoepitope formation. We argue that integrating non-canonical and induced neoantigens into currently available immunotherapy approaches could improve antitumor efficacy and the specificity of resulting therapies, especially in tumors with limited mutational load.

## 1 Introduction

Immunotherapy has revolutionized the treatment of malignant tumors. Unlike traditional approaches such as chemotherapy, radiotherapy, and surgery, which directly target cancer cells and their microenvironment, immunotherapy enhances the patient’s immune response to the tumor or introduces immune cells capable of recognizing and eliminating malignant cells. Although immunotherapy has become a standard of care for several tumor types, many patients do not benefit from treatment while experiencing significant side effects. The success of immunotherapy is associated with several factors, including mutational tumor load (1–3), tumor-infiltrating lymphocytes (TILs) (4–6), and neoantigens (7, 8). This is particularly relevant for tumors with low mutation burden, such as glioblastoma and uveal melanoma, which have shown limited response to current immunotherapies (9, 10).

**Figure 1:**
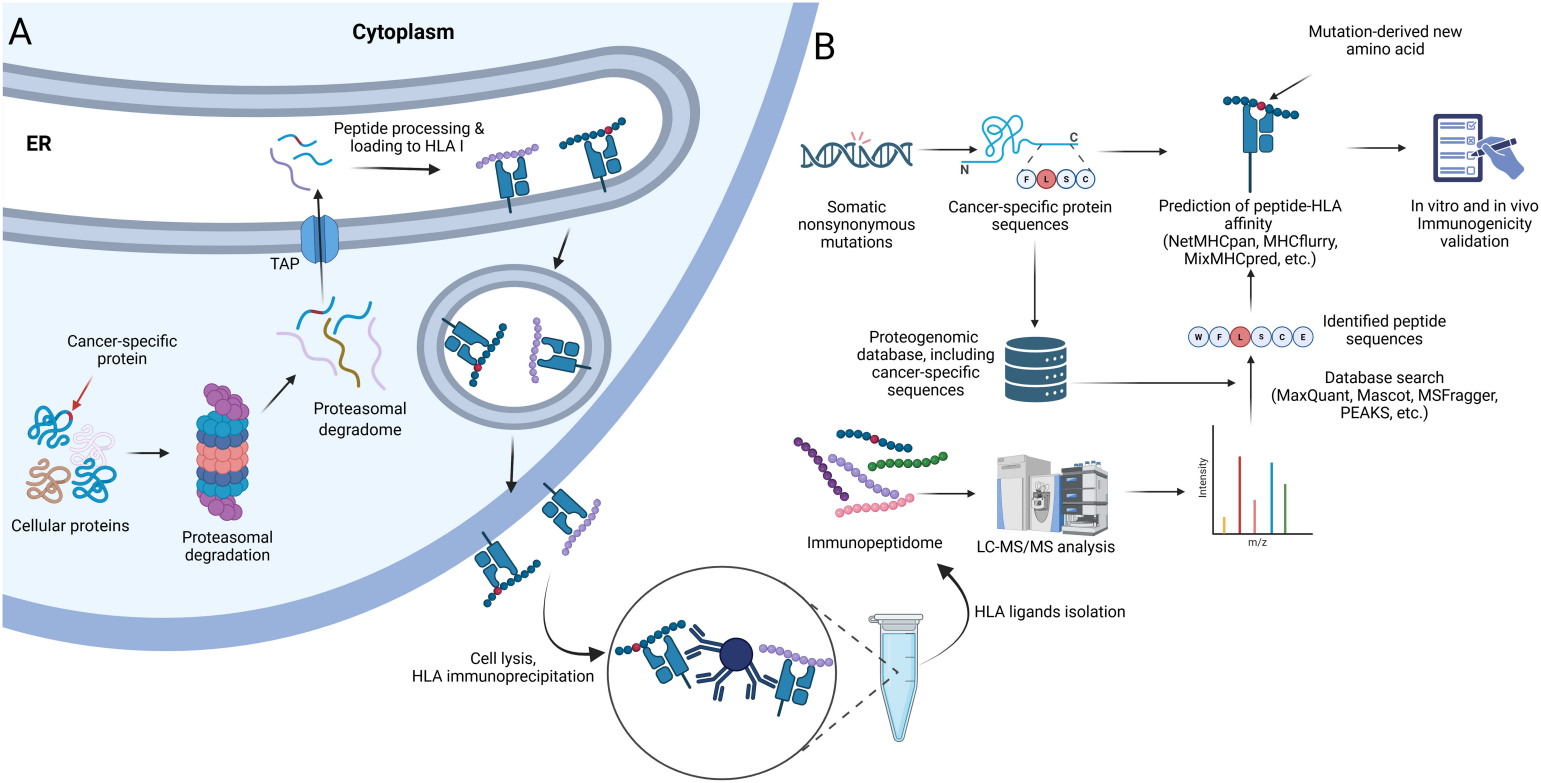
Nature of MHC class I ligands and their identification approaches. **(A)** — Schematic representation of MHC class I ligand presentation pathway. Intracellular proteins, including cancer-specific (indicated by red arrow), undergo proteasomal degradation. Resulting peptides translocate from the cytosol to the endoplasmic reticulum (ER) through TAP (Transporter associated with Antigen Processing), and are loaded into the MHC class I binding groove. The peptide-MHC complexes then traffic to the cell surface. These HLA-peptide complexes can be isolated using immunoaffinity chromatography, and peptide sequences can be determined by mass spectrometry **(B)**; — Typical neoepitope identification pipeline. Tumor-specific DNA mutations are used to predict tumor-specific peptide variants, followed by prediction of peptides binding affinity to the patient’s HLA alleles and subsequent immunogenicity testing. Complementarily, these peptides can be directly observed in the tumor immunopeptidome via mass spectrometry. Importantly, the protein database used to interpret obtained mass spectra should include tumor-specific protein sequences; otherwise, mutation-containing peptides cannot be detected in the mass spectrometry data. Created with Biorender.com.

During the early stages of the T cell–mediated adaptive immune response, recognition of an immunogenic peptide – an epitope – presented by major histocompatibility complex (MHC) class I molecules plays a key role in the development of the immune response. Of particular interest are neoepitopes — immunogenic peptide fragments of tumor-specific antigens (neoantigens) presented on MHC class I (**Figure 1A**). The development of immunotherapies such as immune checkpoint blockade (ICB), cancer vaccines, T cell receptor (TCR) engineered T cell therapy, and chimeric antigen receptor (CAR) T cell therapy depend on neoepitopes, which can be used as a target or as a prognostic biomarker.

Current pipelines for neoepitope identification focus on immunogenic somatic mutations (**Figure 1B**) and have been described extensively elsewhere (11, 12). Briefly, these pipelines search for mutation-derived neoantigens, predict the binding affinity of their peptide fragments to MHC and their immunogenicity, and validate predicted neoepitopes *in vivo* (13–15). Additionally, neoepitopes can be directly identified in the immunopeptidome by mass spectrometry, with subsequent verification of their immunogenicity (16–20). These approaches are complementary and are often used in combination to refine target selection for experimental validation (21). Other techniques, such as ribosome profiling (22) and degradome analysis (23), can further enhance identification reliability. Importantly, mass−spectrometry–based approaches also address the bottlenecks that determine actual antigen presentation – proteasomal processing biases, peptide stability, and HLA−allele restrictions – since not every aberrant mRNA is translated and not every cancer−specific peptide is processed and presented on MHC class I (24). However, despite advances in mass spectrometry and bioinformatics, neoepitope identification remains challenging, limiting the discovery of potential therapeutic targets. This limitation can be overcome by enhancing antigen presentation in tumors (25, 26), allowing the detection of low-abundance neoantigens, or expanding search areas beyond genomic mutations.

Until recently, neoantigens encompassed solely immunogenic non-synonymous genomic mutations in cancer. The term has now broadened to incorporate other cancer-specific antigens derived from RNA modifications, including alternative splicing (16, 27–34) and RNA editing (18, 35, 36), transposable elements (37–39), and other mechanisms (40). Additionally, various approaches can induce neoepitope generation (27, 39, 41–44), including splicing modulation, RNA editing, and epigenetic drugs. Thus, considering neoepitopes derived from diverse sources can expand the repertoire of immunotherapy targets.

This review comprehensively examines current immunotherapy strategies and sources of neoepitopes in cancer – encompassing both naturally occurring and therapy-induced – driven by expanding interest in unconventional tumor antigens. We emphasize advances in splicing-targeted cancer therapies as immunotherapy adjuvants and propose a novel concept: chemotherapy may enhance immunotherapy efficacy by generating splicing-derived neoepitopes through alterations in pre-mRNA splicing.

## 2 Immunotherapy

Advances in our understanding of adaptive immune responses have enabled the development of cancer immunotherapies aimed at enhancing immune-mediated tumor elimination. The concept of cancer immunotherapy has expanded to include diverse strategies, ranging from systemic therapies such as cytokines and immune checkpoint inhibitors (ICI) to targeted approaches involving CAR-based strategies designed to recognize specific antigens and activate immune cells (45). Current immunotherapies can be categorized into five groups: immunomodulators (such as cytokines), monoclonal antibodies, adoptive cell therapies, cancer vaccines, and oncolytic viruses.

Among systemic immunomodulatory approaches, cytokine-based therapies aim to alter immune signaling within the tumor microenvironment. Cytokines are crucial for cell-to-cell communication and immune responses, playing roles in homeostasis, alerting neighboring cells to infections, and recruiting and activating immune cells. Tumors exploit complex cytokine interaction networks to shape an immunosuppressive microenvironment (46, 47). Therefore, modulating cytokine activity – either by enhancing beneficial cytokines or suppressing those exploited by tumors – represents a viable immunotherapy strategy. The application of cytokines in immunotherapy is limited in part by the need for high-dose administration. Although agents such as high-dose IL-2 can induce durable responses in certain cancers, their clinical application is limited by toxicity and modest efficacy (48–50). Furthermore, IL-2 activates regulatory T cells (Tregs) in addition to CD8+ T cells, thereby reducing its therapeutic efficacy (51). For these reasons, cytokines are used primarily in combination approaches or engineered delivery systems (46, 52, 53).

In addition to cytokine-based immunomodulation, monoclonal antibodies represent another major therapeutic class, either by directly targeting tumor-associated surface markers or by modulating immune checkpoints. Monoclonal antibodies target specific surface markers (54, 55) or modulate immune responses, typically acting as ICIs (e.g., anti-PD-1, anti-CTLA-4) that restore T cell activity and have shown clinical success (56–58), particularly in tumors with high tumor mutational burden (TMB) characterized by deficient mismatch repair and microsatellite instability (58–60), or tumors of viral origin (61–63). Identifying neoantigens is therefore crucial for predicting ICB responsiveness. Recently, non-canonical RNA splicing events (neojunctions) have been proposed as additional predictors of response to ICB (30, 64, 65). Emerging antibody formats include bispecific and trispecific antibodies (BsAbs and TsAbs) to further enhance tumor–immune cell interactions (66–68). For example, they can be designed to simultaneously inhibit PD-1 and CTLA-4 signals (68) or to recruit T cells to cancer cells and mediate their contact (66). An important consideration is that because ICBs target molecules crucial for distinguishing between self and non-self, they can cause serious autoimmune side effects (69) that typically resolve upon treatment discontinuation.

Whereas cytokines and checkpoint-targeting antibodies primarily modulate endogenous immune responses, adoptive cell therapies involve the isolation or engineering of immune cells with tumor-directed specificity. Adoptive cell therapies (ACT) include tumor-infiltrating lymphocytes (TILs) and genetically engineered T cells equipped with either a chimeric antigen receptor (CAR-T) or a TCR capable of recognizing specific antigens (TCR-T). These approaches enable specific targeting of tumor antigens and have shown clinical efficacy (70–73). TILs and TCR-T cells recognize intracellular antigens presented by MHC, whereas CAR-T cells target surface antigens independently of MHC (45), which limits the repertoire of CAR targets to surface molecules. Major challenges for ACT include antigen loss and limited efficacy in some tumors (74–78), as well as cross-reactivity against normal tissues when antigens are not cancer-specific (74, 79–81). This underscores the need for cancer-specific neoantigens to avoid potential off-target toxicity.

Another strategy for inducing tumor-specific immunity is therapeutic vaccination, which aims to prime or expand T cell responses against tumor antigens. Cancer vaccines aim to elicit antitumor immunity by delivering tumor antigens using platforms such as peptides (82), nucleic acids (83), dendritic cells (84), whole tumor cells (85), or tumor cell lysates (86) – each characterized by distinct antigen processing efficiency and diversity of epitopes available for T cell recognition. Personalized vaccine strategies have demonstrated promising immunogenicity and durability of T cell responses (21, 87, 88), although their clinical benefit remains variable. Vaccine efficacy – particularly for peptide and nucleic acid platforms – depends critically on the choice of epitopes and on managing neoepitope immunodominance to induce a broad, polyvalent response capable of eliminating tumor cells (89).

Finally, oncolytic viral immunotherapy combines direct tumor cell lysis with immune activation, thereby linking local tumor destruction to systemic antitumor responses. Virotherapy can serve both as a direct mechanism for malignant tumor destruction and as a means of delivering therapeutic molecules into tissues (90, 91). The former approach is more commonly referred to as “oncolytic therapy”, leveraging the capacity of viruses to selectively replicate within tumor cells, ultimately leading to their lysis while leaving normal tissues intact. Upon infection, tumor cells often fail to mount a normal antiviral response, thereby allowing viral replication (91). Replication and subsequent release of new viral particles ensure destruction of surrounding cancerous tissue. Furthermore, when immunogenic cell death occurs, the release of various factors triggers local inflammation (91), thereby converting a “cold” tumor microenvironment into a “hot” one (92). One of the most notable features of virus-based therapies in tumor treatment is their ability to activate both innate and adaptive immunity, thereby promoting a prolonged host immune response (93). For example, Woller and colleagues showed that viral oncolysis evoked cytotoxic T cell responses to a broad panel of neoepitopes (94).

Different immunotherapies are most effective for specific cancer types, and understanding their limitations is crucial for advancing their development. Systemic therapies such as cytokine therapy and ICB can cause autoimmune and other immune-related adverse effects, whereas neoantigen-specific approaches offer greater precision. This highlights the importance of identifying cancer-specific antigens to ensure effective tumor elimination while minimizing harm to healthy tissues. Additionally, the abundance of neoantigens within a tumor serves as a biomarker for predicting responsiveness to therapies such as ICB. Therefore, understanding neoepitope sources beyond non-synonymous mutations is crucial for discovering novel immunotherapy targets.

## 3 Sources of neoepitopes

Cancer antigen classification distinguishes between tumor-specific antigens (TSAs, neoantigens) and tumor-associated antigens (TAAs). While TSAs are usually understood to arise from somatic mutations (canonical neoantigens), an expanding repertoire of non-canonical neoantigens has been identified. These include epitopes derived from alternative splicing, frameshift mutations, viral proteins, non-coding genomic regions (95), transposable elements, protein splicing, and non-canonical translation (23), as well as aberrant post-translational modifications (comprehensively reviewed in (40)).

In contrast to TSAs, TAAs do not generate any novel epitopes but can nevertheless elicit immune recognition and serve as immunotherapy targets. However, TAAs are expressed in normal tissues, typically at lower levels than in tumors, which presents significant limitations. The risk of off-target toxicity (74, 79–81) and reduced immunogenicity due to central tolerance mechanisms make TAAs suboptimal therapeutic targets. Most TAAs represent proteins whose overexpression promotes tumor progression. A notable example is HER2 (*ERBB2* gene), which is overexpressed in ovarian and breast cancers (96, 97).

Within TAAs, two subgroups exhibit more restricted expression patterns in normal tissues, making them relatively more suitable, though still not ideal, immunotherapy targets: differentiation antigens and cancer-testis antigens (CTAs). Differentiation antigens are tissue-specific proteins normally expressed in particular cell lineages. For example, gp100, tyrosinase, and MART-1 are melanocyte-specific proteins involved in melanin biosynthesis (98). As discussed above, targeting TAAs may lead to autoimmune toxicity, thus only antigens with minimal or absent expression in normal tissues are preferable.

CTAs represent another subgroup of TAAs with restricted expression profiles. Their expression is normally confined to immune-privileged sites such as testis and placenta (99), yet they are frequently re-expressed in various malignancies, making them attractive immunotherapy targets. As an example, PRAME, a melanoma antigen, is expressed in a limited number of normal tissues: testis, endometrium, ovary, and adrenal gland (100). Similarly, MAGE antigens demonstrate particularly high expression in melanomas and head-and-neck squamous cell carcinomas (101). Another example is NY-ESO-1, with targeted therapies yielding durable responses in patients with metastatic melanoma and synovial cell sarcoma (102). However, its limited expression across human malignancies restricts broader clinical applicability compared to MAGE-A (103).

**Figure 2:**
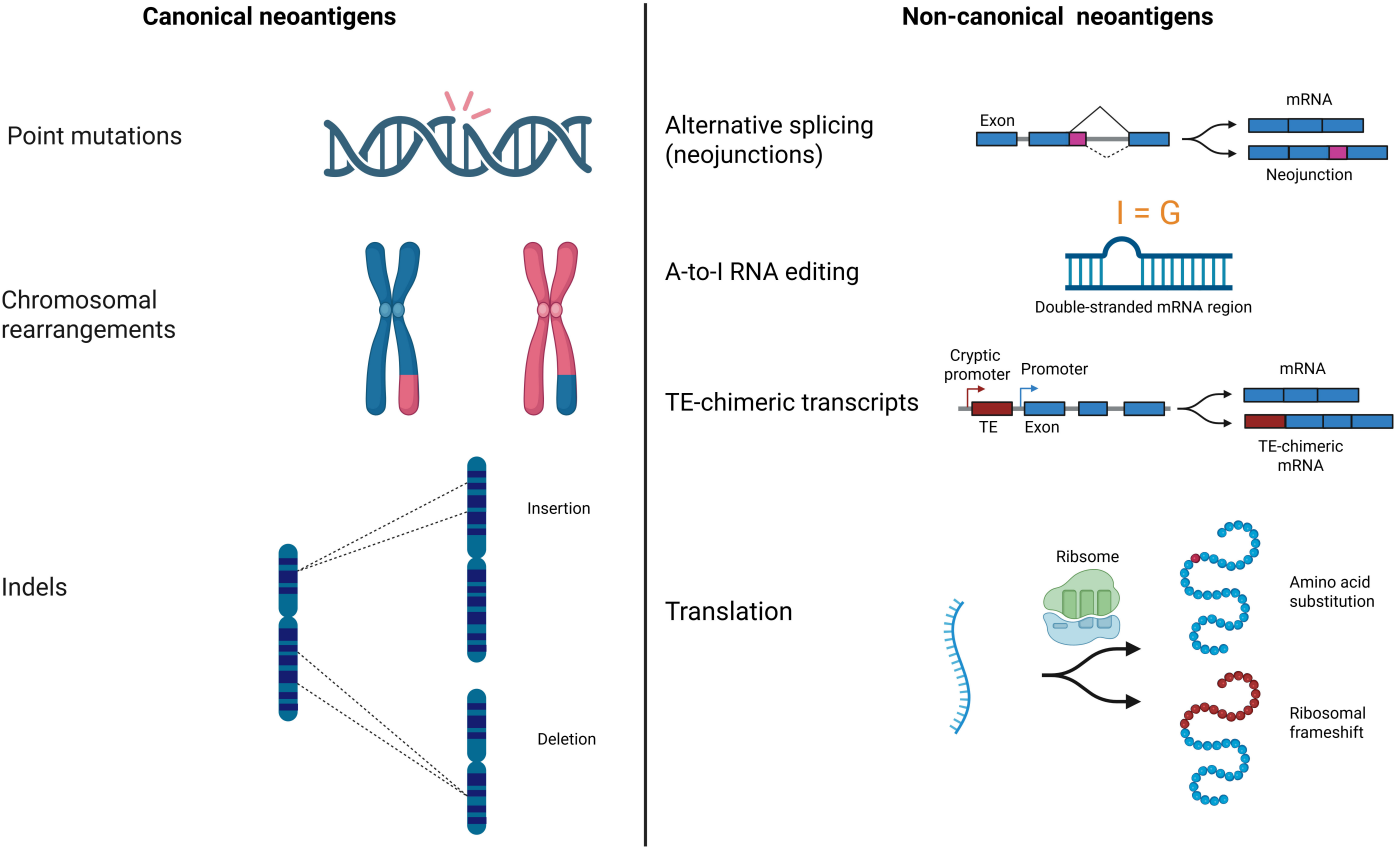
Sources of neoantigens. Canonical neoantigens arise from various genomic somatic mutations, whereas non-canonical stem from alterations during gene expression, including RNA processing (splicing, RNA editing), activation of transposable elements (TE) cryptic promoters, and translation (amino acid substitutions, ribosomal frameshifting). Therapeutic strategies that further disrupt these processes in tumor cells can generate induced non-canonical neoantigens. Created with Biorender.com.

Therefore, immunogenicity alone is insufficient for optimal antigen selection; prevalence across tumor types and expression patterns in normal tissues should be carefully considered to minimize unexpected toxicities (74, 79–81). Given the potential for off-target effects with TAAs, neoantigens – novel antigens absent from normal tissues – represent superior therapeutic targets, minimizing toxicity while limiting off-target effects to the inherent specificity of the targeting technology employed. Of particular interest are neoepitopes – immunogenic short sequences of neoantigens that are presented on cell surfaces in complex with MHC molecules. Neoepitopes can be classified into two categories: naturally occurring neoepitopes, arising from mutations or aberrant cellular processes within cancer cells, and induced neoepitopes, generated in response to cancer therapies (**Figure 2**).

### 3.1 Naturally occurring neoepitopes

#### 3.1.1 Mutation-derived neoepitopes

Cancer-specific genomic alterations – such as single nucleotide mutations, indels, and chromosomal rearrangements – are the most extensively studied source of neoepitopes. Their exclusive presence in malignant cells makes them ideal immunotherapy targets. Among them, driver mutations, which are frequently expressed across the majority of cancer cells within a tumor, are particularly attractive therapeutic targets (104, 105). However, driver mutations may lack immunogenicity or fail to generate peptides that are efficiently processed and presented by MHC molecules, thereby preventing T cell activation.

Despite these challenges, several driver mutations have been successfully targeted for immunotherapy. A prominent example is the substitution of glycine with aspartic acid at position 12 of KRAS (G12D). This hotspot driver mutation generates an epitope recognized by CD8+ T cells, and targeting strategies have shown clinical promise, except in cases where malignant cells have lost MHC class I expression (104). However, the majority of neoantigens derived from mutated proteins are patient-specific (106), substantially limiting their clinical application due to the high cost and technical complexity of personalized neoantigen identification (11).

#### 3.1.2 Splicing-derived neoepitopes

Aberrant splicing is highly prevalent across various malignant tumors and has attracted considerable attention as a source of neoepitopes, particularly in tumors with low mutation burden. Alternatively spliced mRNAs in cancer generate novel proteoforms that can serve as neoepitope sources and contribute to tumor immunogenicity (28–30, 33, 34, 107–112). Notably, alterations in pre-mRNA splicing can produce longer aberrant peptides through the creation of novel junctions (neojunctions), in contrast to the single amino acid substitutions resulting from point mutations.

The first evidence for immunogenic neoepitopes derived from splicing events and capable of inducing T cell response was reported in the late 1990s, with the identification of intron-derived epitopes in melanoma (110, 113). Early transcriptome-based approaches for identifying splicing-derived neoepitopes focused on cancer-specific alternative splicing (AS) events, with validation using available proteomic data and neoepitope prediction algorithms. For example, analysis of splice-site-creating mutations (SCMs) across 8,656 TCGA tumor samples revealed that SCMs can generate neoantigens potentially more immunogenic than those arising from missense mutations. One SCM-derived peptide covering two predicted immunogenic variants was validated in an independent mass spectrometry dataset (107).

A pan-cancer study analyzing 8,705 patients identified an average of 930 splicing neojunctions per patient that were absent in healthy individuals from the GTEx database (108). Importantly, at least one neojunction-derived peptide predicted to bind MHC class I was observed in 43 out of 63 (68%) breast cancer patients, highlighting the substantial potential of splicing-derived neoepitopes for immunotherapy applications. Furthermore, aberrant splicing of splicing factors themselves can lead to widespread splicing dysregulation and potentially facilitate neoepitope generation (109).

Given that cancers harbor novel AS events and that peptides derived from these events can be detected in cancer proteomes, researchers have sought to identify splicing-derived neoepitopes presented on MHC molecules. Smart and his colleagues demonstrated neoepitopes derived from retained introns in the melanoma immunopeptidome (32). Subsequently, T cells reactive to splicing-derived neoepitopes were demonstrated in neuroendocrine prostate cancer, confirming their immunogenicity (33).

Despite the potential of splicing-derived neoepitopes, they may face similar limitations as mutation-derived neoantigens, particularly the challenge of tumor heterogeneity. A critical question is whether recurrent splicing-derived neoepitopes exist that could enable the development of cancer-specific or even pan-cancer vaccines. One study addressed this by exploring whether mutations in the same splicing factor might generate recurrent AS events and, consequently, shared splicing-derived neoepitopes. Indeed, mutations in the splicing factor SF3B1 in uveal melanoma (UM) have been shown to promote the generation of shared splicing-derived neoepitopes, presenting a promising avenue for immunotherapy (29). While this research focused on SF3B1 mutations as drivers of splicing-derived neoepitopes, more recent findings have identified shared neoepitopes in UM patients independently of SF3B1 status (28), expanding potential immunotherapeutic approaches to include SF3B1 wild-type patients. Additionally, overlapping splicing-derived neoepitopes have been observed among melanoma patients (30). Notably, the burden of splicing neoantigens, similarly to TMB, was associated with worse survival in patients who did not receive ICB therapy, while a positive correlation was observed in cohorts treated with checkpoint inhibitors.

Kwok and his colleagues recently conducted a comprehensive analysis of shared splicing-derived neoepitopes, identifying recurrent neojunctions across multiple TCGA patients (34). These neojunctions were not only shared between patients but were also identified across different biopsy sites within individual tumors, demonstrating their tumor-wide presence. These findings were validated using transcriptomic data from various glioma cell lines and proteomic analyses of patient-derived tumors. By integrating tandem mass spectrometry and RNA sequencing data, investigators narrowed the range of putative neoepitopes and focused on peptides predicted to bind strongly to HLA-A*02:01, revealing two immunogenic neoepitopes (NeoA_RPL22_ and NeoA_GNAS_) out of four tested. T cell response to these neoepitopes was dose-dependent, and CD8+ T cells transduced with TCRs specific for these neoepitopes exhibited cytotoxic activity against glioblastoma cell lines. This study also investigated the underlying mechanisms for variability in neojunction frequency across different glioblastoma subtypes, finding that deficiencies in splicing factors and RNA binding proteins contribute to increased neojunction formation. This finding may have implications for other tumor types exhibiting dysregulation in splicing.

Kim and colleagues identified a shared splicing-derived neoepitope present in the tumor stroma of multiple patients and cancer types (16). Their immunopeptidomic analysis revealed a COL6A3 peptide arising from an alternatively spliced proteoform – specifically, an mRNA isoform including exon 6 – expressed in cancer-associated fibroblasts. Furthermore, recurrent splicing-derived neoepitopes and their cognate T cells have been identified in myeloid leukemias (111), highlighting a promising avenue for TCR-T cell therapies in these malignancies.

Collectively, splicing neojunctions represent a previously underexplored source of neoepitopes. Their tumor-wide expression and shared presence across patients suggest considerable potential for immunotherapy applications. Additionally, the burden of splicing neojunctions may serve as a biomarker for predicting immunotherapy responsiveness (112).

#### 3.1.3 RNA editing-derived neoepitopes

Adenosine-to-inosine (A-to-I) RNA editing is a common post-transcriptional RNA modification that occurs in normal cells. This process is catalyzed by adenosine deaminases acting on RNA (ADARs) and primarily prevents unwanted type I interferon (IFN) response to endogenous double-stranded RNAs (dsRNAs) (114). When deamination occurs within exonic sequences, it can lead to non-synonymous amino acid substitution, termed protein recoding, because inosine is interpreted as guanosine by the ribosome (115). However, aberrantly edited transcripts can produce recoded protein isoforms with antigenic properties. Since editing levels at specific sites change during ontogenesis (116) or due to incomplete thymic presentation during T cell selection, RNA editing may create proteoforms and corresponding neoepitopes that elicit T cell responses.

It is now established that RNA editing can generate neoepitopes in cancer and elicit an adaptive immune response. ADAR1 is a member of the interferon-stimulated gene (ISG) family, and since inflammation is a hallmark of cancer (117) and malignant tumors frequently experience interferon-rich microenvironments (118), RNA editing is often upregulated in cancer (119, 120) and can lead to neoepitope formation. RNA editing-derived neoepitopes have been identified using proteogenomic approaches (18, 36). Zhang and colleagues identified a hyper-edited site within cyclin I mRNA in melanoma and confirmed the immunogenicity of the corresponding MHC-I-presented peptide (18). Importantly, this mRNA site is also edited in normal cells; therefore, only increased editing levels can elicit immune responses, categorizing it as a possible TAA. Another peptide derived from oxysterol binding protein-like 9 was identified in the immunopeptidome of ovarian carcinoma (36), although the immunogenicity of this recoded peptide was not established.

Intriguingly, the repertoire of splicing-derived neoepitopes could potentially be expanded by A-to-I RNA editing. RNA editing events predominantly occur in non-coding repeated sequences such as Alu elements (121), which can form dsRNA structures. Since splicing-derived neoepitopes can arise from retained introns (32), which also contain Alu elements, alternatively spliced RNAs with retained introns may be edited by ADARs. This phenomenon has been previously observed in introns retained following *hnRNPC* knockdown (122). Moreover, RNA editing is regulated by splicing efficiency (123). For certain editing sites, the dsRNA structure is formed by exon-intron pairing before splicing. Reduced splicing efficiency extends nuclear residence time for pre-mRNA, increasing the likelihood of editing. This may result in hyper-editing, leading to the generation of novel TAAs as observed with hyper-edited cyclin I (18). Thus, the editing of alternatively spliced mRNAs from which neoepitopes arise presents an interesting perspective on how RNA editing may participate in cellular processes.

#### 3.1.4 Transposable element-derived neoepitopes

Epigenetically dysregulated transposable elements (TEs) represent a hallmark of cancer (124) and can be incorporated into mRNA transcripts, altering their sequence (125). These chimeric RNAs can generate novel sequences presented on MHC class I (37). Studies have demonstrated that tumor-specific, recurrent neojunctions containing TE sequences encode immunogenic peptides (38), and vaccination with these peptides inhibits tumor growth (126). Recently, this discovery has led to the development of TE-based therapies for glioblastoma, where TE-containing transcript generation is induced using the FDA-approved epigenetic drugs decitabine and panobinostat (39).

#### 3.1.5 Translation-associated neoepitopes

As previously discussed, natural neoepitopes arise from perturbations at the DNA or RNA level, or through dysregulation of their processing. However, amino acid substitutions can also occur during translation. For instance, tryptophan and arginine depletion may lead to their replacement by phenylalanine (W>F) and cysteine (R>C), respectively (127, 128). These neoantigens have been termed “substitutants” (127). Besides W>F substitution, tryptophan deficiency can cause ribosomal accumulation downstream of its codon, leading to ribosomal frameshifting events (129). Similarly, leucine deprivation results in ribosomal stalling and consequent formation of neoepitopes derived from aberrantly translated proteins (130).

Another intriguing observation is that amino acid substitutions can occur post-translationally. For instance, the conversion of cysteine to serine in the insulin sequence generates a diabetes-associated neoantigen (131). These translation- and post-translation-derived neoantigens present unique detection challenges because they cannot be predicted from nucleic acid sequencing alone. Their discovery relies entirely on mass spectrometry-based proteomic or immunopeptidomic analysis, although these approaches ultimately depend, at least partially, on nucleic acid-derived information.

### 3.2 Therapy-induced neoepitopes

In addition to neoepitopes arising spontaneously in tumors from genomic mutations or disruptions of cellular processes such as RNA processing, neoepitopes can be generated through external perturbation of cellular processes by therapeutic interventions.

#### 3.2.1 Virotherapy-induced neoepitopes

As previously discussed, oncolytic viruses (OVs) hold significant promise for cancer therapy. Beyond their direct oncolytic properties that facilitate immune targeting of tumors, OVs can serve as delivery vehicles for various genetic or protein payloads (91). Loading OVs with immunogenic proteins or peptides can provide both immunostimulatory functions and enable specific targeting of tumor cells (132). Virus-mediated delivery of neoantigens has demonstrated immunogenicity and antitumor activity in several preclinical and clinical studies. For instance, viral vectors encoding tumor-specific neoantigens elicited immune responses in melanoma mouse models (133) and phase I clinical trials (134). A further refined strategy employs oncolytic adenoviruses for co-delivery of neoantigens alongside pre-assembled peptide-MHC complexes (135), reducing the degree of personalization required since the desired MHC allele is delivered together with the neoepitope.

However, incorporation of exogenous payloads is not the only mechanism by which OVs stimulate the presentation of novel tumor antigens. In a murine ovarian cancer model, reovirus infection significantly altered the tumor immunopeptidome, resulting in the emergence of distinct MHC class I ligands derived from murine proteins (136). Notably, 241 of these virus-induced antigens were absent in control groups treated with IFNγ, despite previous studies indicating that viral infections can reshape the immunopeptidome, potentially through interferon signaling pathways (137).

#### 3.2.2 RNA editing-based neoepitope generation (editopes)

Building upon the RNA-dependent neoepitope generation strategies reviewed by Rosenberg-Mogilevsky with coauthors (42), therapeutic RNA editing presents an attractive alternative approach. This strategy leverages the cell’s endogenous RNA editing machinery for precise, dose-dependent RNA modification, in contrast to genome editing’s irreversible all-or-nothing approach. A key advantage is the relative simplicity of targeting: only a guide RNA is needed (138–140). A preprint by Pecori and colleagues demonstrates the feasibility of employing ADAR to create neoepitopes, specifically through serine-to-glycine (S>G) substitution in MART-1 using antisense oligonucleotides (44). However, this approach is limited to A-to-I(G) modification, restricting its application to certain amino acid substitutions.

#### 3.2.3 Splicing modulator-induced neoepitopes

Splicing modulators represent a class of therapeutic agents that target spliceosome components, typically the SF3B1 complex, or its regulators such as RBM39 or PRMT5. Malignant tumors exhibit heightened sensitivity to spliceosome targeting due to frequent mutations in splicing factors or their regulators (141, 142). Conversely, normal cells remain largely resistant to splicing modulation until they acquire oncologic features such as *MYC* overexpression (143).

Until recently, mechanisms of action underlying spliceosome targeting were poorly understood, but several have been elucidated in recent years (27, 143). These studies revealed that spliceosome targeting engages both innate and adaptive immunity, suggesting potential for enhancing immunotherapy responses. For instance, targeting mRNA splicing can induce formation of splicing-derived neoepitopes (27, 31, 41, 144). Splicing modulation with isoginkgetin derivative IP2, which prevents formation of the pre-spliceosomal A complex, modified the repertoire of tumor-presented peptides and successfully triggered adaptive immune response (41). Another study demonstrated that splicing modulation by RECTAS (rectifier of aberrant splicing) induced neoepitope generation and CD8+ T cell response, enhancing response to ICB therapy (144). Similar results were observed following manipulation of the splicing factor RBM39 and type I PRMT through degradation and inhibition, respectively (27). Additionally, inhibition of protein phosphatases PP2A/PP5, which indirectly affects splicing, generated novel splicing-derived MHC ligands as detected by immunopeptidomics in colon cancer cells (145). Finally, suppression of the splicing factor HNRNPA1 generated splicing-derived neoantigens that reduced tumor growth and increased CD8+ T cell infiltration *in vivo* (31).

Beyond inducing neoepitope formation, splicing modulators have been shown to enhance antitumor responses by recruiting immune cells to the tumor microenvironment. Targeting SF3B1 induced dsRNA-dependent type I IFN response in lung adenocarcinoma cell line A549 and triple-negative breast cancer (TNBC) using pladienolide B (PldB) (146) and H3B-8800 (143), respectively. Moreover, spliceosome-targeted treatment with H3B-8800 enhanced adaptive immune signaling (143). PldB also demonstrated a complex effect on the immune microenvironment in an ovarian cancer mouse model. While it promoted tumor infiltration by both CD8+ and CD4+ T cells and decreased the proportion of regulatory T cells, indicating potential antitumor activity, PldB also increased expression of the immune checkpoint molecule PD-L1 (147). This suggests a potential tradeoff, where PldB’s ability to recruit T cells could be counteracted by PD-L1 upregulation, which may suppress T cell activity and reduce immunotherapy effectiveness. Nonetheless, the combination of PldB and ICB enhanced antitumor response compared to ICB alone (147).

As with direct neoepitope induction, disruption of post-translational modifications of spliceosome components can achieve similar outcomes. For instance, targeting PRMT5 either pharmacologically or through shRNA-mediated knockdown promoted melanoma infiltration by activated CD8+ T cells (148). This indirect spliceosome modulation also enhanced ICB efficacy. Similarly, PRMT1 inhibition successfully increased T cell infiltration and ICB responsiveness in cancer cell lines and TNBC mouse model (149, 150).

Collectively, these studies suggest that splicing modulation can enhance adaptive immune response to tumor through both neoepitope generation and transformation of immunologically “cold” tumors into “hot” ones. Consequently, drugs targeting pre-mRNA splicing may be effectively combined with immune checkpoint inhibitors, although they have not yet been applied as standalone treatments. This approach may yield responses even in tumors previously unresponsive to ICB (107).

#### 3.2.4 Chemotherapy-induced neoepitopes

Chemotherapy encompasses a broad spectrum of cytotoxic molecules that primarily target rapidly proliferating cells. While chemotherapy preferentially affects malignant cells, it also impacts proliferating normal cells, including immune and epithelial cells.

Despite the widespread use of chemotherapy, its impact on neoepitope formation remains underexplored. Differential presentation of MHC class I ligands was observed following doxorubicin treatment *in vitro* (151). Subsequent reanalysis of these data revealed DNA damage-induced MHC ligands derived from transcripts susceptible to nonsense-mediated decay (NMD) (25), although immunogenicity of the discovered peptides was not tested. Furthermore, a recent study demonstrated that chemotherapy can induce neoepitopes derived from upstream open reading frames (152). Thus, these studies provide evidence that chemotherapy is capable of generating neoepitopes from at least two distinct sources.

**Figure 3:**
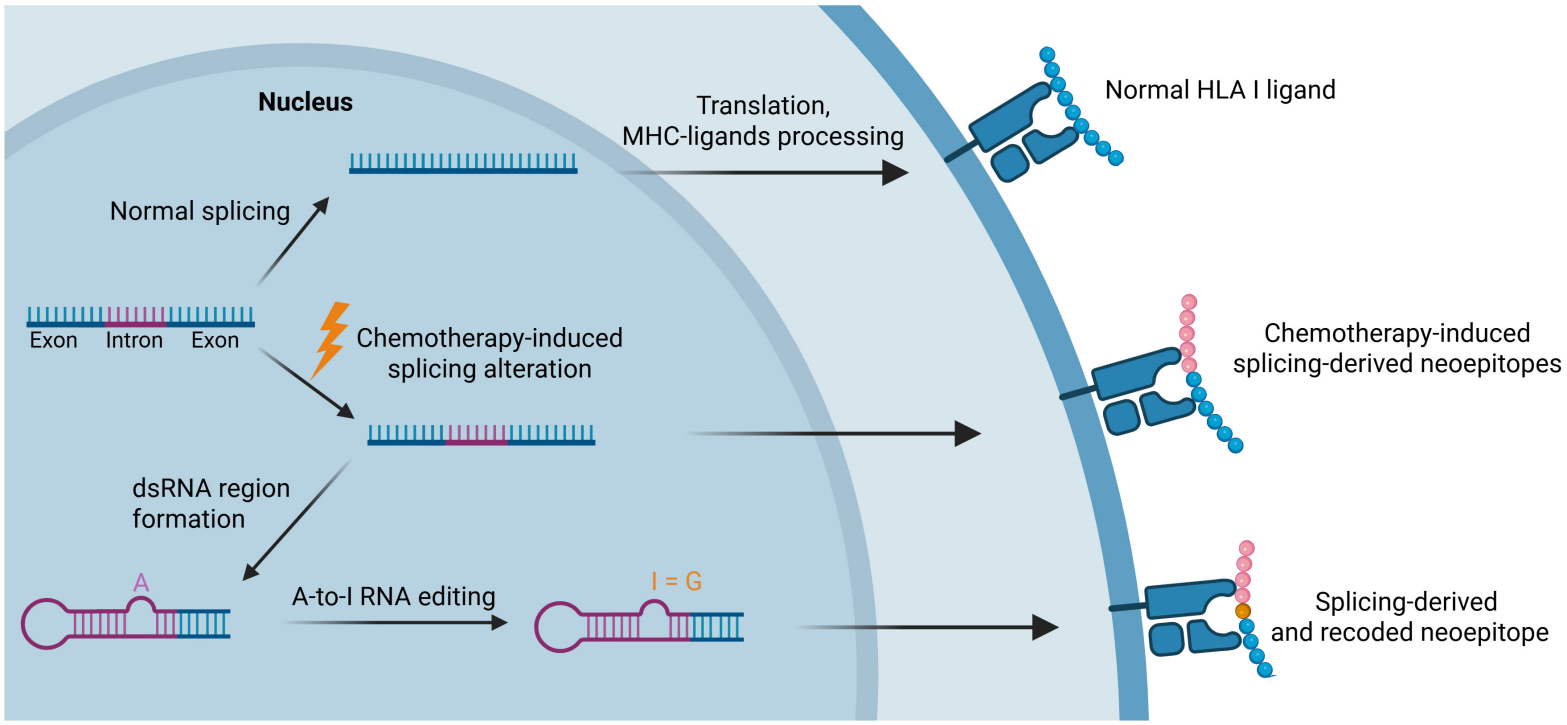
Schematic representation of how chemotherapy may influence the formation of splicing-derived neoepitopes. Normally, pre-mRNA is accurately spliced to produce mature transcripts, and the peptides derived from the resulting protein are processed and displayed on the cell surface through the MHC class I. Chemotherapeutic treatment can disturb normal splicing – for instance, by causing intron retention – resulting in cancer-specific protein variants that may generate new neoepitopes. Furthermore, alternatively spliced transcripts may form double-stranded RNA (dsRNA) regions that become targets for ADAR1-mediated A-to-I editing. When such editing alters the encoded amino acids, it can increase the diversity of these splicing-derived neoepitopes. Created with Biorender.com.

Compelling evidence indicates splicing changes following various types of antitumor chemotherapy (153–155), UV irradiation (156), and hypoxia (157). Since chemotherapy drastically disturbs cellular processes, leading to widespread cell rearrangements, the abundance and activity of splicing factors can change, resulting in altered mRNA splicing profiles (158–160). For instance, chemotherapy-induced tumor extracellular vesicles are enriched in splicing-related proteins. These vesicles promoted aggressiveness in recipient malignant cells through splicing changes in glioblastoma (159) and ovarian adenocarcinoma (158). Thus, chemotherapy-induced alterations in pre-mRNA splicing represent a promising source of novel neoepitopes that could stimulate antitumor immune response (**Figure 3**).

Interestingly, chemotherapy-induced alterations in pre-mRNA splicing exhibit similarities across different agents (155, 160). Analysis of alternative splicing in tumors treated with a broad spectrum of chemotherapeutic agents – including platinum-based agents, paclitaxel, tyrosine kinase inhibitors, and topoisomerase inhibitors, as well as hypoxia and radiation – revealed common patterns in splicing alterations, with intron retention being the most prevalent (160). This suggests that diverse chemotherapeutic agents, despite targeting distinct cellular processes, elicit similar cellular stress responses and mRNA splicing alterations. Notably, these splicing changes were consistent across different cancer types and therapeutic modalities, pointing to the possibility of generating recurrent neoepitopes derived from alternatively spliced RNAs following chemotherapy.

Taken together, splicing-derived neoepitopes generated by chemotherapy could contribute to antitumor immune response, similarly to the effects observed with direct spliceosome modulation (27, 41). Additionally, given that some splicing-derived neoepitopes are shared across patients and tumor types (34), chemotherapy-induced splicing-derived neoepitopes may also prove to be shared between patients, making this type of neoepitopes attractive for broadly applicable immunotherapies.

## 4 Discussion

Current immunotherapy approaches are limited by the need to identify neoantigens, typically derived from non-synonymous genomic mutations, resulting in highly personalized treatments (161). While targeting personalized neoantigens can be beneficial, this approach is costly and time-consuming (11). Moreover, tumor heterogeneity facilitates immune escape from targeted immunotherapies (162). Therefore, homogeneously expressed neoantigens that are shared across patients (also known as public neoantigens) are of greatest interest (163–166). Such neoantigens can make immunotherapies more effective and less patient-specific, thereby reducing production cost and time requirements. However, tumors with low mutation burden present challenges for neoantigens discovery, limiting immunotherapy applications. Thus, expanding the repertoire of potential neoantigen sources may be beneficial.

Considerable effort has focused on neoantigens arising from non-synonymous genomic mutations, whereas the potential of unconventional neoantigens – such as those derived from aberrant mRNA splicing and RNA editing – has been relatively underexplored but is now gaining increasing attention (35, 42, 167). Notably, to our knowledge, no studies have investigated the potential for RNA editing to diversify the neoepitope repertoire arising from alternatively spliced RNAs (**Figure 1**). This represents a potentially significant knowledge gap regarding the interaction of these two processes in generating novel targets for immune surveillance.

Non-canonical neoepitopes do not, by themselves, imply superior immunogenicity to canonical neoepitopes or increased sharing between patients. Rather, incorporating them expands the repertoire of known neoepitopes, some of which may prove to be more effective as therapeutic targets or prognostic biomarkers in the context of immunotherapy. The primary benefit lies in increasing the pool of potential targets, rather than suggesting that one class of neoantigens is inherently superior to another; however, this remains to be further investigated. To this end, all classes of non-canonical and induced neoepitopes should be described in greater detail. Following their identification and confirmation of immunological activity, key features such as temporal stability, clonal persistence, and reversibility after treatment withdrawal must be systematically evaluated. This is essential to determine whether these neoantigens remain therapeutically targetable for a sufficient duration to support vaccine- or TCR-based approaches.

Splicing-modifying drugs can generate neoepitopes, including shared and tumor-wide ones, but none are FDA-approved for cancer treatment. Indirect splicing modulators such as PRMT5 and RBM39 inhibitors are currently in phase I-II clinical trials (NCT03573310, NCT05245500, NCT05094336, NCT05528055, NCT05024994), while trials of the promising direct spliceosome inhibitor RVT-2001 (H3b-8800) were discontinued in 2024.This setback highlights the continued need for research and development of spliceosome-targeting therapies, both as standalone treatments and in combination with immunotherapies. As splicing-modulating drugs remain primarily in preclinical stages or early-phase clinical trials, exploring chemotherapy as a potential source of splicing-derived neoepitopes for immunotherapy represents a promising avenue.

Chemotherapy can sensitize refractory tumors to ICB (168), with combination therapy demonstrating superior efficacy to chemotherapy alone (169, 170). Furthermore, while ICB is effective in deficient mismatch repair colorectal cancer (CRC) (59, 60), it is ineffective in CRC with proficient mismatch repair (60) unless combined with chemotherapy (171, 172). This chemotherapeutic priming of immunotherapy has been previously reviewed (173).

We propose that, in addition to other known mechanisms, chemotherapy’s contribution to immunotherapy efficacy may include alterations in splicing and consequent neoepitope generation. These neoepitopes, generated through RNA splicing alterations, could potentially enhance the efficacy of existing immunotherapies by creating new targets for immune recognition and elimination, as observed with targeted spliceosome modulation or inhibition of splicing regulators (27, 31, 41, 144). The consistent splicing changes observed across various tumor types treated with different chemotherapy agents (160) may suggest the generation of a shared repertoire of chemotherapy-induced splicing-derived neoepitopes. This is particularly attractive because many novel immunotherapies enter clinical trials for treating therapy-refractory cancers with poor prognosis. Thus, utilizing neoepitopes generated by chemotherapy offers a promising strategy for developing anticancer vaccines or ACTs, especially in tumors with low mutation burdens. For instance, several studies have identified TCRs specific to shared splicing-derived neoepitopes (16, 33, 34), which hold promise for TCR-T cell therapy development. This approach could significantly expand the number of targetable neoepitopes and consequently improve immunotherapy efficacy, which currently benefits only a limited subset of patients (174–178).

Although the concept of chemotherapy-induced splicing-derived neoepitopes is intriguing, its implementation faces several limitations. As with other MHC-dependent strategies, a key limitation is the ability of tumors to evade immune recognition through downregulation of MHC class I processing and presentation machinery. In such cases, CAR-T cell therapy targeting splicing-derived surface neoantigens could be developed as an alternative approach. Another concern is that transcripts containing retained introns are typically targeted by NMD. However, studies have demonstrated that immunogenic neoepitopes can arise from alternatively spliced RNAs (27, 41), suggesting that these RNAs can bypass NMD or that NMD is ineffective, which is frequently observed in tumors (179). Furthermore, chemotherapy can decrease NMD efficiency (180). Additionally, neoepitopes can be generated from alternatively spliced RNAs containing premature stop codons through the pioneer round of translation (25, 181). Immunologically “cold” tumors pose another challenge. However, independent studies have shown that splicing modulation in various tumor types can increase immune cell infiltration and enhance ICB efficacy (27, 41, 147–150). Intratumoral heterogeneity may limit the fraction of tumor cells that present chemotherapy-induced neoepitopes. However, this limitation is not specific to the proposed approach; rather, it represents a common challenge for most targeted and antigen-specific cancer therapies. Importantly, chemotherapy-induced neoepitopes may arise in a substantial fraction of treated tumor cells, rather than being restricted to a pre-existing minor subpopulation. Moreover, the proposed strategy is intended primarily as part of combination therapy, in which elimination of neoepitope-positive cells may complement the cytotoxic effects of chemotherapy and potentially induce secondary antitumor immune responses through epitope spreading. Finally, targeting splicing machinery either through splicing drugs or chemotherapy carries the risk of affecting normal cells and potentially generating splicing-derived neoepitopes in non-malignant tissues. However, disruption of splicing in normal cells should be limited, as therapeutic doses typically affect normal cells to a lesser extent (182, 183). Additionally, treatment with cancer cell secretomes collected after chemotherapy did not affect splicing in fibroblasts (158), and direct splicing modulation did not induce intron retention in normal cells (143). Besides the mechanistic limitations discussed above that affect neoantigen generation, chemotherapy−induced adaptive transcriptomic responses can also promote immune escape, increased antigenic heterogeneity, dedifferentiation, or activation of adaptive suppression pathways (184, 185). Because chemotherapies are already clinically used, these risks do not preclude their study, but they raise an important question: to what extent can chemotherapy be an effective inducer of clinically meaningful neoepitopes?

The identification of peptides presented in complex with MHC molecules is more challenging than analyzing standard proteomic data. Conventional proteomic approaches employ highly specific proteases (typically trypsin) that cleave protein sequences at defined sites, such as after arginine and lysine residues, enabling straightforward *in silico* database generation (186). However, MHC-presented peptides lack consistent cleavage patterns, complicating bioinformatic analysis and hindering comprehensive immunopeptidome characterization. To address this obstacle, various methods have been developed to enhance detection yields (187–189). Another important consideration is neoepitope identification. While standard proteomic searches utilize databases generated from *in silico* translation of the reference genome, neoepitope identification requires a more personalized approach. Proteogenomics employs databases constructed from individual patient genomes and/or transcriptomes, incorporating specific mutations, rare alternative splicing events, or RNA editing events not present in standard proteomic databases (35). This personalized approach enables detection of unique neoepitopes arising from individual genetic or mRNA variations. Initially employed for identifying cancer neoantigen peptides in tumor proteomes, proteogenomics has since expanded to include searches for peptides recoded by ADAR (190) and neoantigens generated through RNA editing (18, 35, 36), as well as splicing-derived neoepitopes (27, 32–34). Importantly, this approach relies on incorporating known alternative splicing and RNA editing events into *in silico* predictions of novel peptides. However, current workflows for discovering alternative splicing or RNA editing events face limitations (191), often restricting analyses to previously established events.

In conclusion, the systematic identification of neoepitopes from diverse sources – both naturally occurring and therapy-induced – is essential to fully characterize the tumor neoepitope repertoire. This will expand the pool of potential immunotherapy targets, potentially enabling selection of those that are more tumor-specific, shared across patients, and stably expressed. Priming tumors with chemotherapy represents a potentially significant yet understudied source of splicing-derived neoepitopes. While challenges remain, the potential benefits warrant further investigation of this hypothesis in future studies of chemotherapy-treated tumors.
